# Relationship between complement and macrophage markers with kidney survival in patients with diabetic nephropathy

**DOI:** 10.1007/s00592-025-02521-3

**Published:** 2025-05-08

**Authors:** Ozcan Uzun, Cihan Heybeli, Fatma Sema Anar Kutlu, Manolya Celebioglu Pekiner, Filiz Yıldırım, Caner Cavdar, Sulen Sarioglu

**Affiliations:** 1 Yalova Research and Training Hospital, Yalova, Turkey; 2 Division of Nephrology, Dokuz Eylül University School of Medicine, Izmir, Turkey; 3 Department of Pathology, Dokuz Eylül University School of Medicine, Izmir, Turkey; 4 Division of Nephrology, Ege University School of Medicine, Izmir, Turkey; 5 Zonguldak Atatürk State Hospital, Zonguldak, Turkey

**Keywords:** Complement system proteins, Diabetes mellitus, End-stage kidney disease progression, Macrophages, Renal failure

## Abstract

**Background:**

Diabetic nephropathy (DN) is the leading cause of end-stage kidney disease (ESKD) worldwide. Macrophages and the complement system have interrelated roles in DN. We aimed to determine associations between macrophage and complement markers with the progression of DN.

**Methods:**

This retrospective cohort study included patients diagnosed with sole DN by kidney biopsy. Using immunohistochemistry, CD68^+^ and CD163^+^ cells and complement markers were counted in glomerular and tubulointerstitial areas. The primary outcome was evolution to ESKD and/or doubling serum creatinine (SCr).

**Results:**

Forty-six patients were included. The median SCr at baseline was 2.7 (1.41–3.1) mg/dL. During the median follow-up of 32 months (range 6–54), 50% of patients reached the primary outcome. Most of the clinical and histological findings were comparable between progressors and non-progressors, while progressors had a higher median number of glomerular CD68+ cells and a higher percentage of glomerulosclerosis. After adjustments for age, sex, and SCr, the median glomerular CD68+ cell number was the sole independent predictor of progression. Glomerular C4d was associated with nephrotic-range proteinuria but not with the progression of kidney failure.

**Conclusions:**

Glomerular CD68+ cell count may serve as a promising predictor of kidney disease progression among patients with DN. Glomerular C4d was associated with nephrotic-range proteinuria but not with the progression of kidney failure.

## Key points

- ∙ Many patients with diabetic nephropathy progress to renal failure and experience diabetic nephropathy-related mortality despite strict hemodynamic and metabolic control.
- ∙ Targeting inflammation has been proposed as a new therapeutic approach for diabetic nephropathy.
- ∙ Glomerular CD68+ cell count predicts the development of ESKD and/or doubling serum creatinine (SCr) in patients with diabetic nephropathy.

## Introduction

Diabetic nephropathy (DN) remains the most common cause of end-stage kidney disease (ESKD) globally [1]. The main initiator of DN is chronic hyperglycemia, while various pathological mechanisms such as hemodynamic disruption, oxidative stress, inflammation, and fibrosis play a role in the deterioration of renal function [2]. A significant proportion of DN patients develop renal failure and DN-related mortality despite strict hemodynamic and metabolic control [3, 4]. In addition to the treatment of hemodynamic and metabolic pathways, targeting inflammation has been proposed as a novel therapeutic approach for DN [5]. High glucose and advanced glycation end products activate adhesion molecules in the vascular endothelium, subsequently promoting circulating monocytes [6, 7]. Synthesis of chemokines in glomerular podocytes, mesangial cells, and renal epithelial cells leads to intrarenal migration of macrophages and inflammation [8, 9]. The production of macrophage colony-stimulating factor-1 from renal parenchymal cells causes both the migration of more monocytes from the circulation to the damaged tissue and the proliferation of resident macrophages [10]. The diabetic milieu increases pro-inflammatory cytokine production, reactive oxygen, matrix metalloproteases, mitogenic growth factors, tissue factors, and profibrotic cytokines from macrophages [11, 12]. Uncontrolled macrophage activation results in inflammation or the development of fibrosis in the glomeruli and tubulointerstitial areas of the kidney.

Macrophages are central players in inflammation. Two types of macrophages have opposing features. M1 macrophages (classical activation) cause high microbial activity and tissue damage after interferon-gamma (IFN-γ) and lipopolysaccharide stimulation. In contrast, M2 macrophages (alternative activation) are stimulated by interleukin-4 and interleukin-13, leading to the secretion of growth cytokines, downregulation of inflammation, and wound healing [13]. Klessens and Nguyen demonstrated the presence of macrophages in both glomeruli and interstitium at all stages of DN [14, 15] and macrophages have roles both during the development and the progression of DN [16]. Macrophages play a crucial role in chronic inflammation, one of the characteristics of diabetes and diabetic nephropathy [16]. Wang and colleagues evaluated the effects of activated versus resting macrophages using an experimental one experimental mouse model of focal segmental glomerulosclerosis in adriamycin-nephrotic mice with severe combined immunodeficiency [17]. The authors observed increased inflammation and damage in kidneys exposed to activated macrophages and decreased kidney function but not in mice exposed to macrophages in a resting state [17]. Macrophages may lead to kidney damage by producing reactive oxygen species, cytokines, and proteases [12]. Depending on their polarization, macrophages can either exacerbate renal inflammation or contribute to renal repair. Although the roles of macrophages in the development and progression of diabetic nephropathy are known, the relationship between macrophage phenotypes in the clinical context is unclear.

Another crucial part of the host defense is the complement system, which is composed of a complex network of plasma and membrane-associated serum proteins [18]. Macrophages and the complement system cooperate closely [19–21] and macrophages bear complement receptors. Phagocytosis, an important mechanism of the host-defense system and primary function of macrophages, is facilitated by opsonization, a process by which serum components tag pathogens for recognition by neutrophils and macrophages [22]. Similar to macrophage markers, some studies reported associations between complement products and the severity and progression of diabetic nephropathy [23, 24]. Based on these, we aimed to investigate the relationship between histological markers of macrophages and complement proteins, their interactions, and their role in progression to ESKD among patients with DN.

## Materials and methods

### Patient population and study design

This study is a retrospective cohort study of patients with type 2 diabetes diagnosed with DN via renal biopsy in a tertiary care hospital between January 2000 and October 2019. Among the 51 patients evaluated, hemodialysis was initiated in 5 patients at presentation to reduce bleeding complications of kidney biopsy and for volume control. These five patients were excluded from the analysis since they remained dialysis-dependent during follow-up. We obtained the data from medical files and electronic medical records.

Exclusions were:

- < 18 years of age.
- Coexistent diagnosis in kidney biopsy (including acute tubular injury, acute interstitial nephritis, IgA nephropathy, etc.)
- Inadequate biopsy samples.
- Follow-up of < 6 months.
- Acute disease within the previous 1 month of kidney biopsy (infection, heart attack, acute kidney injury, etc.)
- Systemic autoimmune conditions, malignancy, and drug exposure may cause a change in phenotype in macrophages (immunosuppressive therapies).

### Outcomes

The date of renal biopsy was the baseline, and we gathered the laboratory data from the closest time. We defined ESKD as a permanent need for renal replacement therapy and/or an estimated glomerular filtration rate (eGFR) of < 15 ml/min/1.73 m^2^. We calculated the estimated GFR using the CKD-EPI formula [25]. The last visit date or the date of the decision to initiate a chronic renal replacement therapy option was accepted as the final date. Follow-up time is the period between the final date and the date of the kidney biopsy. The study’s primary outcome was evolution to ESKD and/or doubling serum creatinine.

### Histopathologic evaluation

We obtained histopathological data from previous pathology reports. Glomerular lesions, vascular lesions, and interstitial lesions were scored according to the established histopathological classification for DN [26]. Tissue adequacy criteria were accepted as ≥ 8 glomeruli for light microscopy and ≥ two glomeruli for immunofluorescence microscopy. Absence of interstitial fibrosis and tubular atrophy (IFTA) is scored as 0 (zero) points; 1 point is given for < 25% IFTA; more than 25% but less than 50% IFTA as 2 points; >50% IFTA is scored as 3 points and considered severe [26]. 

## Immunohistochemistry

**Macrophage markers** CD68+ cells represent pan-macrophages, and CD163+ cells represent M2 (anti-inflammatory) macrophages. Immunohistochemical staining was performed on consecutive sections of tissue samples from cases of DN. Monoclonal mouse antibodies against CD68 (Cell Marque, CD68 [Kp-1] Mouse Monoclonal Antibody, Catalog no. CMC16829040) and Monoclonal mouse antibodies against CD163 (LEICA/Novocastra, CD163 [10D6] Monoclonal Antibody, Catalog no. PA0090) were used.

The number of CD163-positive and CD68-positive cells in the glomerular area was the number of positive cells per glomerulus. The number of cells in the tubulointerstitial area was the number of positive cells per high-power field. We analyzed five fields and calculated the mean number of cells. We evaluated the cortical compartment only since only a subgroup of patients had adequate medullary biopsy. One investigator who was unaware of the clinical data performed the histopathological evaluation.

**C4d** The paraffin-embedded block sections were incubated for 52 min in 95 °C CC1 (cell conditioning 1) solution (Ventana Medical Systems, Tucson, Arizona, USA) and then for 32 min in diluted primary monoclonal anti-C4d antibody (Cell Marque, SP91 clone, Rocklin, CA). We used the Ultraview Universal DAB Detection kit (Ventana Medical Systems, Tuscon, Arizona, USA) for stainings in the Benchmark Ultra equipment. Positive controls included tonsillectomy and membraneous nephropathy specimens. We used light microscopy to assess the biopsies and glomerular C4d immunohistochemistry staining to classify the samples as positive or negative. C4d was identified as positive in at least one glomerular region. The interlobular capillaries, tubular epithelium, and afferent arteriole were also evaluated. At least three nonsclerotic glomeruli were required to assess glomerular C4d staining.

**Data availability** The data associated with the paper are not publicly available but are available from the corresponding author on reasonable request.

### Statistical analysis

We presented categorical variables as numbers and percentages. We presented continuous variables as median and the interquartile range (25–75%) if the variables were not distributed normally and as mean and standard deviation in the case of normal distribution. We performed the chi-squared tests for comparisons between categorical variables and the Mann-Whitney U test for comparisons between continuous variables. We used Cox regression analysis to determine predictors of the progression of kidney disease. Results are expressed as hazard ratios (HR). Age, Sex, baseline serum creatinine, histologic grade, and rate of glomerulosclerosis were included in the multivariate regression model, along with macrophage counts. We performed sample size and power calculations to evaluate the statistical robustness of our study investigating the relationship between glomerular M1 macrophages and ESKD. We conducted a power analysis using G*Power statistical software with parameters set for a two-tailed test (α = 0.05, 1-β = 0.80). The analysis revealed that to detect an effect size (Cohen’s d = 0.8), a minimum total sample size of 52 participants would be required. Our study, comprising 46 participants, achieved 80% power (95% CI: 69.0–90.9%) to detect large effect sizes at α = 0.05. Post-hoc power analysis confirmed that the study maintained sufficient power (β > 0.80) for detecting large effect sizes, though it may be underpowered for identifying more subtle clinical differences. We used SPSS (IBM SPSS, Chicago, IL) version 22.0 for the statistical analysis. P values less than 0.05 were regarded as statistically significant.

## Results

### General characteristics

Among 51 patients, we excluded five because they remained dialysis-dependent after the kidney biopsy. Of the 46 patients, 25 (54%) were male, and the mean age was 55 ± 13. The median time from the diagnosis of diabetes mellitus to kidney biopsy was 14 (1-30) years. The median serum creatinine and eGFR were 2.7 (1.41–3.1) mg/dL and 32 (21–51) ml/min/1.73 m^2^. Twenty-two (48%) had an eGFR of < 30 ml/min/1.73 m^2^ at presentation. Nephrotic syndrome was evident in 50% of patients. Indications for a kidney biopsy included nephrotic syndrome (50%), absence of typical chronology of diabetic nephropathy (46%), and active urine sediment (4%).

### Histological findings

**Fig. 1:**
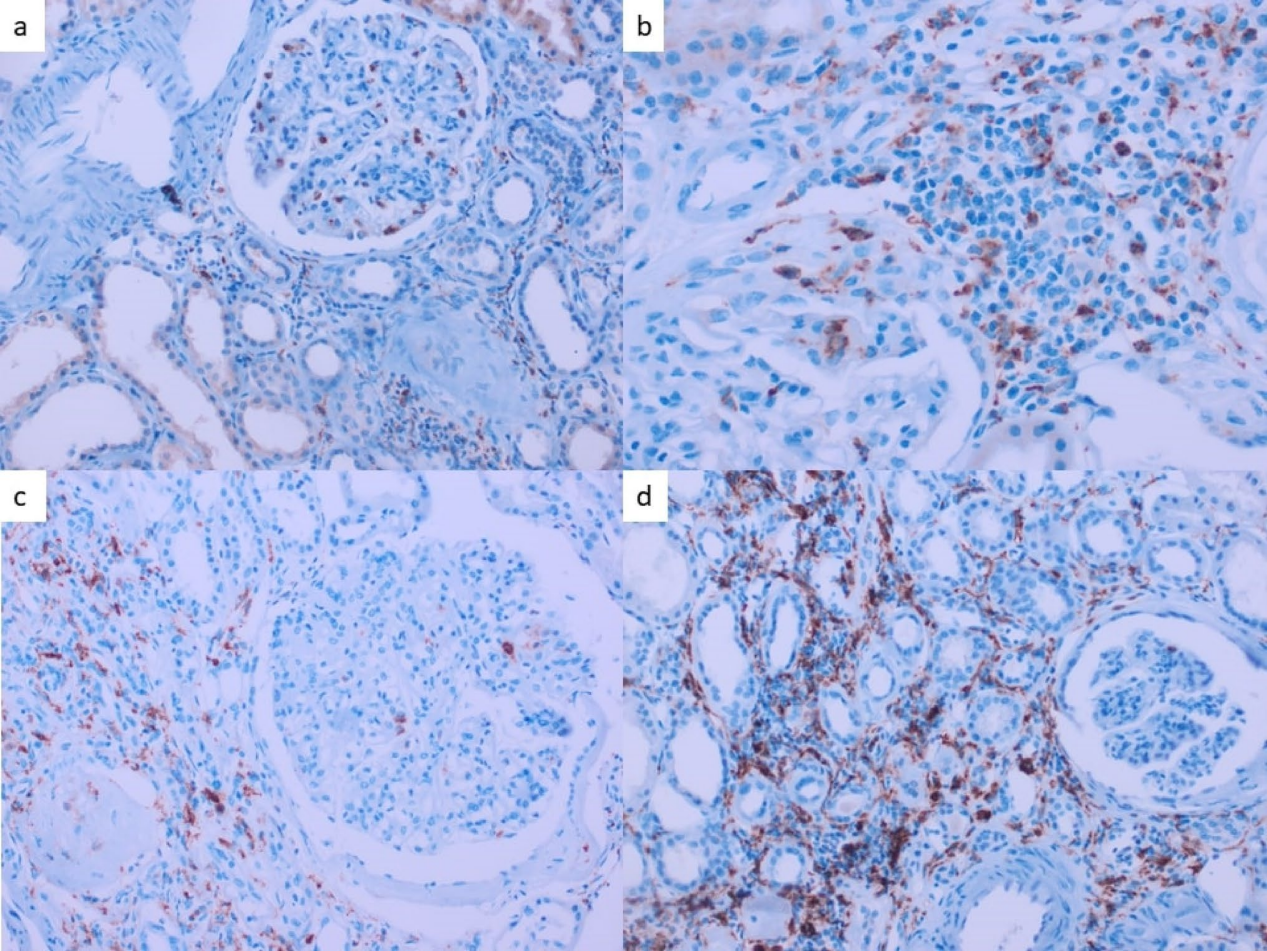
Immunohistochemical evaluation of CD68+ and CD163+ Cells in Renal Tissue (a) CD68+ cells in glomerular regions, demonstrating macrophage infiltration. (b) CD68+ cells in interstitial regions, highlighting tubulointerstitial macrophage. (c) CD163 + cells in glomerular regions, indicating M2 macrophage distribution. (d) CD163+ cells in interstitial regions, showing M2 macrophage localization. We obtained all images using immunohistochemical staining (IHC) at an original magnification of ×40

Due to technical issues, we could not evaluate glomerular CD68+ cells in two patients and CD163+ cells in three. The tubulointerstitial count was unavailable for CD68+ cells and CD163+ cells in two patients and one patient, respectively. For glomerular cell count, the median number of CD68+ and CD163+ cells were 1.6 (0.5–2.9) and 0.6 (0–1.1) per glomerular area, respectively (Fig. 1). The median number of CD68+ and CD163+ cells for tubulointerstitial area were 3.4 (2–5.5) and 6.7 (3.5–10.2) per high power field, respectively.

### Follow-up and outcomes

During the median follow-up of 32 months (ranging from 6 to 54 months), 23 (50%) of patients had progression (evolution to ESKD or doubling of serum creatinine). The only significant difference between the progression and non-progression groups was the median number of glomerular CD68+ cells among numerous demographic, clinical, and histological markers (Table 1). Median glomerular CD68+ cell count was 2.3 (0.8–3.4) in the progression group, while it was 1 (0–2.4) in the non-progression group (*p* = 0.023).

**Table 1:** Characteristics of patients with diabetic nephropathy who developed end-stage kidney disease versus those who did not

Variables	Progressors (*n* = 23)	Non-progressors (*n* = 23)	*p*-value
Age	54 ± 11	56 ± 14	0.644
Female sex	43%	47%	0.767
Body-mass index, kg/m^2^	27.4 ± 3.9	25.1 ± 4.9	0.445
Insulin therapy	82%	69%	0.300
RAS inhibitors	78%	69%	0.502
Blood pressure, mm Hg			
Systolic	146 ± 22	146 ± 22	0.859
Diastolic	84 ± 10	83 ± 12	0.917
*Laboratory work up*			
Serum creatinine, mg/dl	2.13 (1.51–3.30)	2.20 (1.30–3.00)	0.423
eGFR, ml/min/1.73 m^2^	32 (16–56)	32 (19–62)	0.560
Hemoglobin, g/dL	10.5 ± 1.2	10.8 ± 1.8	0.937
Serum albumin, g/dL	3.2 ± 0.5	3.3 ± 0.7	0.475
HbA1-c, %	7.4 ± 1.5	7.7 ± 1.5	0.510
Proteinuria, g/24 hours	4.6 (2.7–9.8)	4.4 (1.4–8.9)	0.974
Nephrotic syndrome	60%	39%	0.140
Glomerular hematuria	43%	33%	0.490
C3, mg/dl	114 (106–138)	119 (109–140)	0.545
C4, mg/dl	29 (26–35)	34 (25–40)	0.306
Total cholesterol, mg/dl	208 ± 70	205 ± 53	0.707
LDL-cholesterol, mg/dl	121 ± 46	122 ± 38	0.842
Triglyceride, mg/dl	154 (134–284)	202 (130–282)	0.944
HDL-cholesterol, mg/dl	44 ± 12	46 ± 13	0.770
Uric acid, mg/dl	7 ± 1.4	6.7 ± 1.8	0.455
*Histopathology*			
CD68^+^glomerular	2.3 (0.8–3.4)	1 (0–2.4)	**0.023**
CD163^+^glomerular	0.4 (0–1.3)	0.6 (0–1.1)	0.805
CD68^+^interstitial	4 (3–6)	3.5 (1–5.4)	0.099
CD163^+^interstitial	6 (3.8–12)	7 (2.9–10.1)	0.820
CD68^+^ total	6.6 (4.3–9.6)	4.5 (1.8–7.9)	0.076
CD163^+^ total	6.8 (4.6–13)	7.6 (3.9–11.7)	0.733
CD68^+^/CD163^+^ glomerular	2 ((1.6–5.3)	2.1 (0–2.9)	0.233
CD68^+^/CD163^+^ interstitial	0.6 (0.4–1.0)	0.5 (0.3–0.8)	0.264
CD68^+^/CD163^+^ total	0.8 (0.6–1.2)	0.7 (0.4–1.4)	0.418
Macrophage total	16.7 (10.1–21.3)	13.5 (7.8–17.0)	0.239
Complement C4d	39%	48%	0.474
Immunofluorescence microscopy			
IgM	57%	39%	0.232
IgG	33%	39%	0.690
C1q	14%	4%	0.252
C3	33%	39%	0.690
GS over 50%	40%	26%	0.292
GS over 25%	59%	39%	0.181
GS%	41 (18–53)	18 (11–50)	0.183
Severe IFTA	39%	34%	0.672
Diabetic nephropathy stage			n/a
1	0	2	
2	5	7	
3	9	8	
4	9	5	
Diabetic nephropathy stage 3–4	78%	56%	0.165

Bold values represent statistically significant (*p* < 0.05) differences between groups. We presented continuous variables as mean ± standard deviation for normally distributed data and median with the range for non-normal distribution

### Predictors of progression

In the univariate analysis, therapeutic regimens, including the use of renin-angiotensin-aldosterone system (RAAS) blockers and insulin medication, as well as clinical conditions (hyperlipidemia, obesity, smoking, and family history of diabetes), had no significant effect on the type or number of glomerular macrophages. However, the median number of glomerular CD68+ cells (HR 1.27, 95% CI 1.03–1.57, *p* = 0.029) and percentage of glomerulosclerosis (HR 9.25, 95% CI 1.23–69.37, *p* = 0.030) were predictors of progression (Table 2). We included age, sex, baseline serum creatinine, glomerular CD68+ cell number, and percentage of glomerulosclerosis in the multivariate regression model. After adjustments, the median glomerular CD68+ cell number (HR 1.25, 95% CI 1.00–1.55, *p* = 0.050) was the sole independent predictor of progression. There was a trend toward significance for the association between the percentage of glomerulosclerosis and progression (HR 8.72, 95% CI 0.97–78.8, *p* = 0.054). Neither serum levels nor histological stains of complement products were associated with progression.

**Table 2:** Predictors of progression of kidney disease in diabetic nephropathy

	Univariate			Multivariate		
	HR	95% CI	p	HR	95% CI	p
Age	1.00	0.97-1.04	0.981	1.01	0.97-1.05	0.726
Female sex	0.89	0.38-2.07	0.779	1.19	0.46-3.09	0.727
Hyperlipidemia	1.21	0.53-2.77	0.652	-	-	-
Obesity	1.23	0.13-11.87	0.859	-	-	-
Smoking	2.32	0.58-9.28	0.235	-	-	-
Family history of diabetes	2.27	0.56-9.24	0.254	-	-	-
ACEi/ARB	1.27	0.46-3.50	0.642	-	-	-
Insulin therapy	1.61	0.54-4.78	0.393	-	-	-
Serum creatinine (baseline)	1.39	0.94-2.05	0.101	1.41	0.92-2.16	0.116
Proteinuria	0.93	0.84-1.03	0.169	-	-	-
Nephrotic syndrome	1.23	0.51-2.92	0.647	-	-	-
HbA1-c	0.90	0.68-1.19	0.462	-	-	-
Hematuria	0.96	0.41-2.27	0.929	-	-	-
Serum uric acid	1.14	0.86-1.53	0.368	-	-	-
Serum C3	1.00	0.98-1.01	0.816	**-**	**-**	**-**
Serum C4	0.99	0.94-1.03	0.515	**-**	**-**	**-**
CD68^+^glom	1.27	1.03-1.57	0.029	**1.25**	**1.00-1.55**	**0.050**
CD68+ int	1.01	0.92-1.11	0.772	-	-	-
CD68+ total	1.04	0.96-1.12	0.351	-	-	-
Advanced histologic stage (3-4)	1.19	0.42-3.38	0.744	-	-	-
Complement C3 deposition	0.63	0.25-1.59	0.324			
C4d deposition	0.60	0.25-1.45	0.259	-	-	-
Glomerulosclerosis, %	9.25	1.23-69.37	0.030	**8.72**	**0.97-78.8**	**0.054**
Severe IFTA	1.27	0.53-3.07	0.592	-	-	-

ACEi/ARB: Angiotensin-converting enzyme inhibitor/angiotensin receptor blocker

### Clinical and histological correlations between cell counts in glomerular and tubulointerstitial areas

**Fig. 2:**
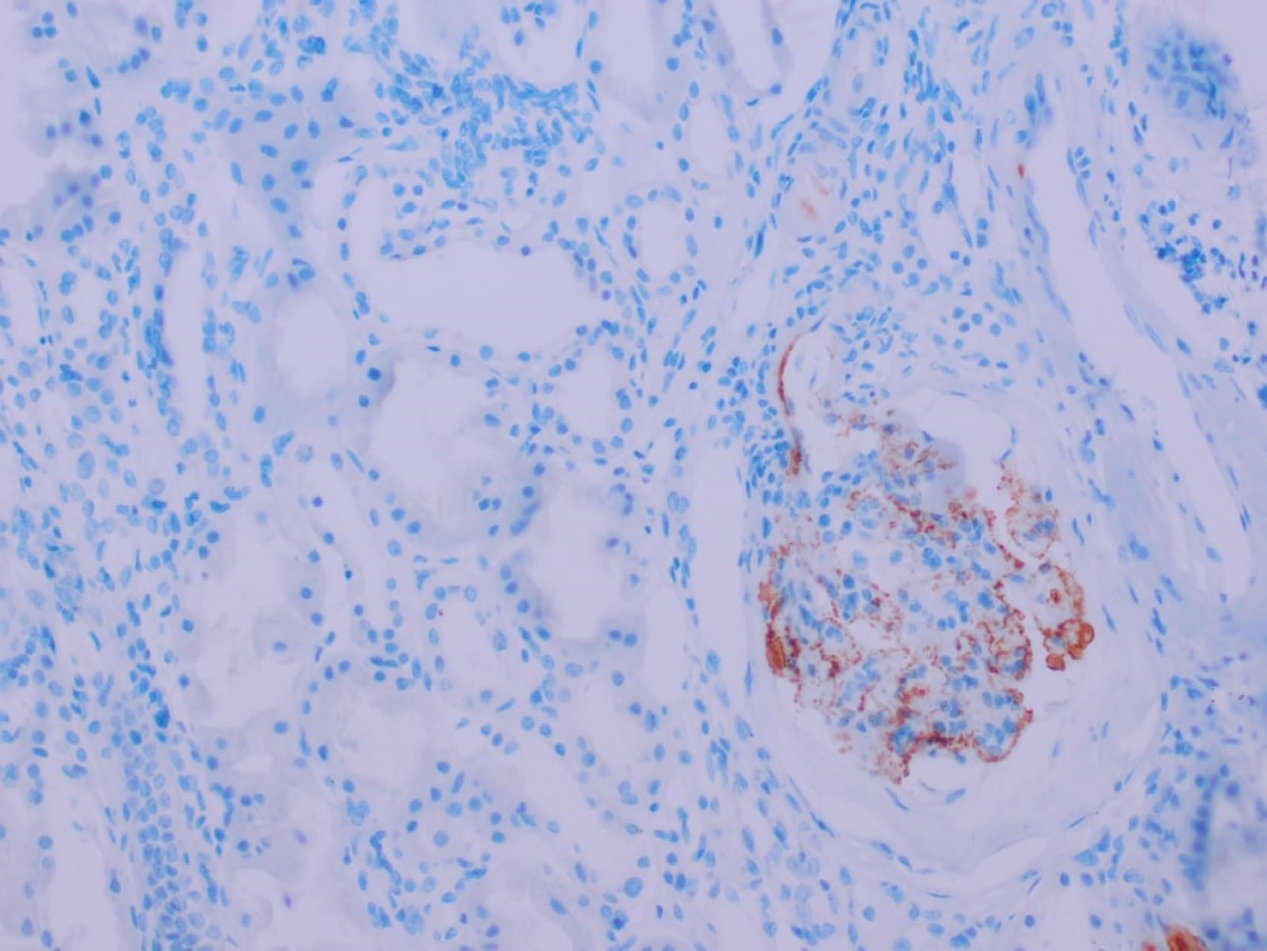
Glomerular C4d Positivity IHC staining demonstrating C4d deposition in glomerular structures. Image captured at an original magnification of ×40

We classified biopsies according to macrophage counts in glomeruli and interstitial areas as ≥ median versus < median. Clinical characteristics and histological findings of patients were compared (Table 3). Based on this classification, patients with higher levels of glomerular CD68+ more commonly had severe interstitial fibrosis/tubular atrophy (*p* = 0.030), higher median number of interstitial CD68+ cells (*p* = 0.007), and higher median number of CD163+ cells in the glomerulus (*p* = 0.019). Patients with a higher glomerular CD163+ cell count had higher serum C4 levels (*p* = 0.002). Patients with higher tubulointerstitial CD68+ cell counts also had higher glomerular CD68+ cell counts (*p* = 0.006) and tubulointerstitial CD163+ cell counts (*p* = 0.044). A higher tubulointerstitial CD163+ cell count was associated with more glomerular CD163+ cells (*p* = 0.025) and interstitial CD68+ cells (*p* = 0.039). Higher CD163+ cells in the tubulointerstitium were associated with a higher prevalence of C4d positivity (Fig. 2). However, this association was not statistically significant (*p* = 0.08).

**Table 3:** Comparison of clinical and histological characteristics of patients with sole diabetic nephropathy stratified by glomerular and tubulointerstitial macrophage counts

	Glomerular							Tubulointerstitial					
	CD68+ cells ≥ median	CD68+ cells < median	*P*	CD163+ cells ≥ median		CD163+ cells < median	*P*	CD68+ cells ≥ median	CD68+ cells < median	*P*	CD163+ cells ≥ median	CD163+ cells < median	*P*
*Demographic, clinical*													
Age, years	54 (41–61)	56 (47–67)	0.357	53 (47–62)		56 (43–66)	0.808	54 (47–63)	56 (45–67)	0.641	53 (42–60)	60 (50–70)	**0.026**
Serum creatinine, mg/dl	2.43 (1.50–3.29)	2.17 (1.38–2.82)	0.370	2.28 (1.35–3.22)		1.99 (1.50–3.15)	0.990	2.51 (1.51–3.30)	1.99 (1.20–2.92)	0.165	2.60 (1.50–3.24)	2.13 (1.41–2.95)	0.759
Proteinuria, g/day	5.8 (1.5–9.8)	4.5 (2.3–4.3)	0.803	7.7 (3.3–10.3)		3.9 (2.9–8.7)	0.290	4.5 (2.7–6.6)	4.9 (2.2–9.7)	0.909	4.9 (3.1–8.7)	4.6 (1.3–10.1)	0.838
HbA1-c, %	7.7 (6.3–8)	7.4 (6.8–9.3)	0.349	7.8 (6.9–9.2)		7.3 (6.4–8.6)	0.251	7.4 (6.9–8.3)	7.6 (6.3–9)	0.791	7.4 (6–8.6)	7.6 (7.1–8.8)	0.379
C3, mg/dl	118 (110–144)	114 (108–139)	0.657	119 (110–144)		113 (87–144)	0.518	114 (109–140)	118 (110–140)	0.873	115 (109–144)	116 (91–139)	0.782
C4, mg/dl	33 (26–44)	31 (25–39)	0.790	35 (28–45)		27 (19–30)	**0.002**	29 (25–36)	35 (29–40)	0.343	33 (26–40)	31 (24–37)	0.518
*Histopathology*													
Diabetic nephropathy stage III-IV	81%	59%	0.099	81%		60%	0.118	79%	60%	0.165	73%	66%	0.599
Severe IFTA	54%	22%	**0.030**	42%		38%	0.753	47%	28%	0.190	47%	28%	0.190
Glomerulosclerosis ≥50%	45%	22%	0.112	42%		28%	0.334	39%	28%	0.460	41%	25%	0.246
C4d, %	52%	35%	0.258	54%		33%	0.161	41%	37%	0.708	56%	30%	0.081
CD68+ cells													
Glomerular	-	-	**-**	2.3 (1.4–3.3)		0.8 (0.3–2.6)	**0.033**	2.4 (0.9–3.2)	1 (0–2.1)	**0.006**	2.1 (0.5–2.9)	1 (0.4–3.3)	0.732
Interstitial	4.2 (2.8–6.6)	2.4 (2.4–3.4)	**0.007**	3.3 (0–16)		3.8 (2.2–6.5)	0.884	-	-	-	4.4 (2.8–6.6)	2.5 (1.6–3.8)	**0.039**
CD163+ cells													
Glomerular	1 (1–1.4)	0.1 (0.1–0.9)	**0.019**	-		-	**-**	0.6 (0–1.4)	0.6 (0–1.1)	0.442	1(0.3–1.3)	0.1 (0.1–0.8)	**0.025**
Interstitial	7.8 (7.8–12)	5.3 (5.3–8.1)	0.091	8.5 (4.1–12.1)		6.0 (2.7–8.4)	0.130	7.4 (4.5–12.2)	5.6 (2.5–8.4)	**0.044**	-	-	-

## Correlation between complement and macrophages

The clinical findings showed a significant association between nephrotic-range proteinuria and C4d deposition in glomeruli. Nephrotic-range proteinuria was evident in 59% of patients with positive C4d versus 14% in patients with negative C4d (*p* = 0.006). There was no significant difference between early (grade I-II) and late (grade III-IV) diabetic nephropathy regarding complement studies in serum or kidney biopsy. As given in previous sections, there was no significant association between serum complement levels, the deposition of complement products, and the progression of kidney disease. Of note, when the five patients initially excluded from the analysis due to dialysis dependency after the kidney biopsy were included, we found a significant association between the number of glomerular CD163+ cell counts and C4d positivity. (median 1.06 versus 0.25 cells per glomerulus in C4d-positive and C4d-negative groups, respectively, *p* = 0.049)

## Discussion

Diabetic nephropathy is a chronic inflammatory condition characterized by the involvement of macrophages [27]. Relationships between macrophage phenotypes and the severity of DN have been demonstrated, but the relationship between macrophage types and evolution to ESRD in the pure DN group is unclear. This study demonstrates that glomerular CD68+ macrophage infiltration, identified by kidney biopsy patients with pure DN, is an independent predictor of ESRD progression during longitudinal follow-up with extensive clinical data. Prior investigations have predominantly relied on autopsy-derived data [14] or included heterogeneous cohorts without a rigorous exclusion of confounding factors such as secondary renal pathologies (e.g., coexisting glomerulonephritis) or non-diabetic disease [28] contributing to macrophage activation. In this cohort of diabetic nephropathy patients undergoing tissue biopsy, glomerular CD68+ cells predicted the development of ESRD even in advanced renal failure.

Progressive renal impairment is associated with macrophage infiltration into the renal cortex [29]. Nguyen et al. found a correlation between baseline serum creatinine and glomerular macrophages [15]. Zhang et al. demonstrated an association between glomerular CD68+ and kidney functions [28]. They observed macrophages in the glomeruli even in the early stages (I + IIa) of patients with DN, and the M1/M2 ratio was highest in the early stage, implying an inflammatory burden. In approximately one-third of patients with DM, the onset of DN may be due to inflammation triggered in the acute phase. Acute renal damage causes inflammation, eventually damaging the kidneys and increasing fibrosis, resulting in long-term functional deterioration. There is strong evidence for a progression from acute renal injury to chronic renal failure [30]. Diabetic nephropathy can lead to renal failure within years, with a complex interplay between hyperglycemia, metabolic factors, and inflammation. Macrophages have crucial roles in inflammation and healing. However, an excess of cells may be a sign of inflammation and/or fibrosis, which may accelerate the progression of kidney disease.

Kidney biopsy in patients with DM may help identify a diagnosis other than DN requiring a specific therapy for the suspected condition. Pathological features of sole DN are descriptive rather than predictive, and they do not provide additional benefits for treatment options. There is a need for markers to predict ESRD to shed light on new treatment approaches in diabetic nephropathy. Including information about macrophage markers in the pathological examination of DN may give more information. Macrophage density can help to classify patients into risk groups for evolution to ESRD. Similarly, research studies predicting response to immunosuppression in IgA glomerulonephritis showed that patients with higher glomerular CD68+ infiltrate density were 13 times more likely to respond to immunosuppression [31]. Inflammation has a role in the progression of DN and may be a treatment target. Inhibition of the mobilization of inflammatory cells into the kidney was protective in experimental DN [32]. Inhibitors of chemokine receptors improved proteinuria in the setting of diabetic nephropathy in mice [33] and humans [34]. A higher number of glomerular CD68+ cells despite an advanced stage of DN might indicate ongoing inflammation in these patients.

Gene expression and cell number profiles differ between early and late DN [35]. Both Zhang and Nguyen’s studies demonstrated higher numbers of glomerular CD68+ cells in earlier stages [15, 28]. Compared to previous studies, the median CD68+ glomerular cell count was lower in our cohort (Table 4). Our patients had a longer duration of diabetes, worse kidney function during biopsy, and higher histological stages. Chronicity in our patients may explain the lower median number of macrophages in the glomerular area. Interestingly, the sole independent predictor of evolution to ESKD was the glomerular CD68+ cell count in our study. Reduced cellularity in advanced DN may be typical due to chronic changes, but even in this cohort, a higher number of CD68+ macrophages in a cohort of advanced DN may still be significant, as this increase represents inflammation [36]. 

**Table 4:** Comparative summary of studies on macrophage association in diabetic nephropathy

	Our study	Klessens [14]	Zhang et al. [28]	Nguyen [15]
Sample Size (N)	46	88	46	20
Age (years)	55 ± 13	70.6 ± 10.8	Not reported	49 (19.0–79)
Female Sex (%)	46%	46.4%	Not reported	40%
Serum Creatinine (mg/dL)	2.7 (1.41–3.1) mg/dL	1.84 ± 0.13 mg/dL	0.99 mg/dL	1.31 ± 1.33 mg/dL
Proteinuria (g/24 hours)	Median 4.5	Present in 40% of patients	Median:3.4	Mean 3.2
Stage 3–4 DN (%)	67%	43.1%	48%	Not reported
Diabet Duration (years)	14 (1–30)	12.7	Not reported	6.5
Glomerular CD68+ Macrophages (glom/gcs)	1.6 (0.5–2.9)	Mean 4.2 (0–19) Median 2.9	2.7 ± 1.1	2.8 + 0.7
Glomerular CD163+ Macrophages (glom/gcs)	0.6 (0–1.1)	Mean 2.1 (0–14.47) Median 1.6	Not reported	Not reported
Interstitial CD68+ Macrophages	3.4 (2–5.5)/gcs	Semiquantitative evaluation 0 = 10% under 1 for 10–30% 2 for 30–50% 3 more than 50%	CD68-positive cells (%)/interstitium 0.039 ± 0.014	296.9 + 63.3/mm^2^
Interstitial CD163+ Macrophages	6.7 (3.5–10.2)	Not reported	Reported as percentage	Not reported
Follow-up (months)	32 (6–54)	Autopsy Findings	Not reported	60
ESKD (%)	50%	Not reported	Not reported	Not reported
Aim	Association between macrophages in kidneys with progression to ESKD	Correlations between macrophage counts with clinical and histological characteristics of diabetic nephropathy	Macrophage phenotype and its relationship to renal function and histological changes in human DN and the effect of triggering receptor expressed on myeloid cells (TREM) on high-glucose-induced macrophage activation	Correlations between macrophage counts with clinical course of diabetic nephropathy
Finding	Glomerular CD68+ count is an independent predictor of ESKD	Interstitial CD68+ macrophages correlate with glomerular filtration rate stage and albuminuria	Positive correlation between M1/M2 activation status and DN progression, and TREM-1 plays an essential role in high glucose-induced macrophage phenotypic change	Macrophages accumulate in glomeruli and interstitium in diabetic nephropathy; the intensity of interstitial infiltrate is proportional to the rate of renal function decline
Limitations	*Most of the patients had severe diabetic nephropathy *Available follow-up data	*Autopsy study *No longitudinal data on macrophage association with ESKD progression	*No longitudinal data on macrophage association with ESKD progression *Macrophage counts evaluated in small subgroups (*n* = 6 each)	*Small sample
Strengths	*Pure diabetic nephropathy confirmed by biopsy in all cases * Follow-up data available with macrophage counts correlated with primary outcomes	*Relatively larger sample size *Clinical– histological correlations available	*Macrophage expression profiles analyzed using in vitro studies *Clinical– histological correlations available	*Long follow-up period (> 5 years in 90% of cases)

(**Abbreviations**; ESKD, End-stage kidney disease; Gcs, glomerular cells)

Determination of macrophage phenotype in the setting of irreversible structural damage in advanced diabetic nephropathy may not be straightforward. On the other hand, many studies confirmed a correlation between macrophage numbers and the extent of kidney damage, the accumulation of interstitial matrix proteins, and the severity of interstitial fibrosis [8, 9, 15]. Although effects of the diabetic milieu on glomeruli are better recognized, diabetes mellitus may also have an impact on the tubulointerstitium [37]. In most comparisons, even though not statistically significant, chronicity findings were more evident among patients with a cell count greater than or equal to the median in both glomerular and tubulointerstitial areas. Another factor that may mask a possible direct relationship between macrophages and diabetic nephropathy is the severity of kidney dysfunction. A cohort study involved seventeen disease entities and found that reduced eGFR and ESKD risk increased with increased CD68+-macrophage density in the cortex, medulla, and whole renal tissue [38]. Another study showed that cortical CD68+ macrophage density was associated with both patient and graft survival and delayed graft function in early protocol biopsies taken 6 weeks after transplantation [39]. These findings may suggest a role for macrophages in the progression of kidney damage, regardless of the underlying cause.

Several studies have shown associations between products of the complement system and clinical or histological grades of DN. However, studies on the relationship between the complement system and macrophage subtypes in DN are lacking. Duan et al. found that serum C4 levels were positively associated with proteinuria, the rate of progression to ESRD, and doubling serum creatinine [40]. In an autopsy study, Bus et al. evaluated kidney biopsy sections for complement stains in patients with diabetes mellitus. Patients with diabetic nephropathy had a significantly higher prevalence of glomerular C4d deposition than patients with diabetes mellitus without nephropathy (73% vs. 60%, *p* < 0.001) [23]. It is unclear if this study included patients with only diabetic nephropathy. The study by Li and colleagues found a correlation between urinary complement proteins and kidney functions and histological grade of diabetic nephropathy [24]. We could not demonstrate a significant association between serum complement levels or glomerular deposition of C4d and C3 with outcomes. One reason for this may be that DN classes were not homogeneous because of the design of our study, as most of the cohort consisted of patients with severe nephropathy. Our median GFR was around 32 ml/min and was lower than those reported in studies by Bus and Li (mean GFR 54 and 44 ml/min, respectively). Given the active role of the complement system in the early stages of DN, it is possible that this contradiction occurred because severe renal failure affected over half of the individuals in this research. It is also worth noting that there is no direct evidence to demonstrate that the complement system has a primary role in the pathogenesis of diabetic nephropathy; instead, it is a secondary driver of the progressive damage [41]. Finally, immunohistochemistry was performed in paraffin-embedded tissues, where complement antigens required careful antigen-retrieval procedures due to the masking of antigens during processing into paraffin. Thus, false negative results are possible [42]. We selected using C4d as a method to determine complement activation to overcome this problem. C4d remains detectable even after a long time due to its ability to bind to cell surfaces covalently [43]. This makes C4d the footprint of immune damage [43]. Still, complement-related damage may be overlooked since C4d represents activation of the lectin and classical pathway but not the alternative pathway [43]. The significant association between glomerular C4d and nephrotic-range proteinuria likely reflects the immune damage to the glomerular area. The lack of significance between C4d and the progression of kidney disease may be explained by proteinuria being only one of several drivers of kidney disease progression in diabetic patients. Furthermore, factors such as the severity of kidney disease and metabolic acidosis can all alter complement levels [44]. 

### Limitations and strengths of the study

Due to our study’s retrospective design, it is impossible to determine a cause-effect relationship. The time of biopsy indication depended on the clinical presentation of the patients; therefore, the comorbid diseases, duration and severity of DM, and treatments differed between the patients. However, we excluded those who recently received immunosuppressive therapies. Another concern may be the relatively small sample size, which limits the opportunity to detect significant associations in subgroups. While the inclusion of patients confirmed by biopsy is a strength, the results may not apply to all patients with diabetic nephropathy since most of our patients already had a progression of kidney disease to some extent. The median GFR of our cohort was around 32 ml/min/1.73 m^2^, and the risk of evolution to ESKD is very high in such patients with diabetes, limiting the generalizability of our findings to all patients with diabetic nephropathy; however, studies that provide information about macrophage profiles and complement split products in severe DN are lacking, and these findings may suggest further hypotheses for subsequent studies in these patients. Higher glomerular CD68+ cells, even in advanced stages of DN, might indicate an ongoing inflammation in these patients. Our study is unique in that we carefully excluded all possible coexistent kidney disorders. We analyzed associations between macrophage counts in the kidney and the progression of kidney disease after a median follow-up of 32 months. Due to their close relationship in the disease pathogenesis, we examined the histological and clinical impact of the complement system and macrophages. Further studies are needed to delineate the possible relationship between the complement system and macrophages during DN.

## Conclusions

Glomerular CD68+ cell count may predict the development of ESKD and/or doubling serum creatinine (SCr) in patients with DN. Glomerular C4d was associated with nephrotic-range proteinuria but not with the progression of kidney failure. Our findings support the possible predictive role of macrophage markers in the progression to ESKD in patients with DN. Glomerular CD68+ cell counts could be promising predictors of kidney disease progression among patients with DN and may shed light on new treatment pathways.

## Data Availability

The data supporting this study’s findings are available from the corresponding author upon reasonable request.
